# Spontaneous Intracranial Hemorrhage: A Sign of Cavernous Angioma Diagnosis in Pediatric Age Group

**DOI:** 10.7759/cureus.14917

**Published:** 2021-05-09

**Authors:** Sayed Mohammed Jawad Alwedaie, Meysam Abolmaali

**Affiliations:** 1 Department of Neuroscience, Salmaniya Medical Complex, Manama, BHR

**Keywords:** cerebral cavernous malformation, developmental venous anomaly, paediatric age

## Abstract

Cerebral cavernous malformation (CCM) is a developmental abnormality of blood vessels that supply the brain. It is composed of large, adjacent capillaries which contain little or no neural tissue. They mostly occur in the supratentorial region. However, the occurrence of these vascular lesions can be seen at different sites of the central nervous system (CNS). The prevalence of CCM is estimated to be 0.4% in the general population and among the affected patients, 18.7% have multiple lesions. However, about 30-50% of CCM cases are asymptomatic and are found incidentally. Here we report a case of an eight-year-old girl with a massive hemorrhagic presentation of a left parietooccipital CCM.

## Introduction

Developmental cerebrovascular malformations are types of brain vascular anomalies, which consist of endothelial-lined caverns. Any defect on endothelial tight junctions will make this vascular malformation more susceptible to leak or bleed. The prevalence of this vascular anomaly is estimated to be between 0.4% and 0.8%; however, it has a rare occurrence in the pediatric age group [1] and few studies have been published about symptomatic cerebral cavernous malformations (CCMs) till now. In this report, we are going to discuss a case of CCM in a pediatric patient with serious symptoms and recurrent cerebral hemorrhage.

## Case presentation

**Figure 1:**
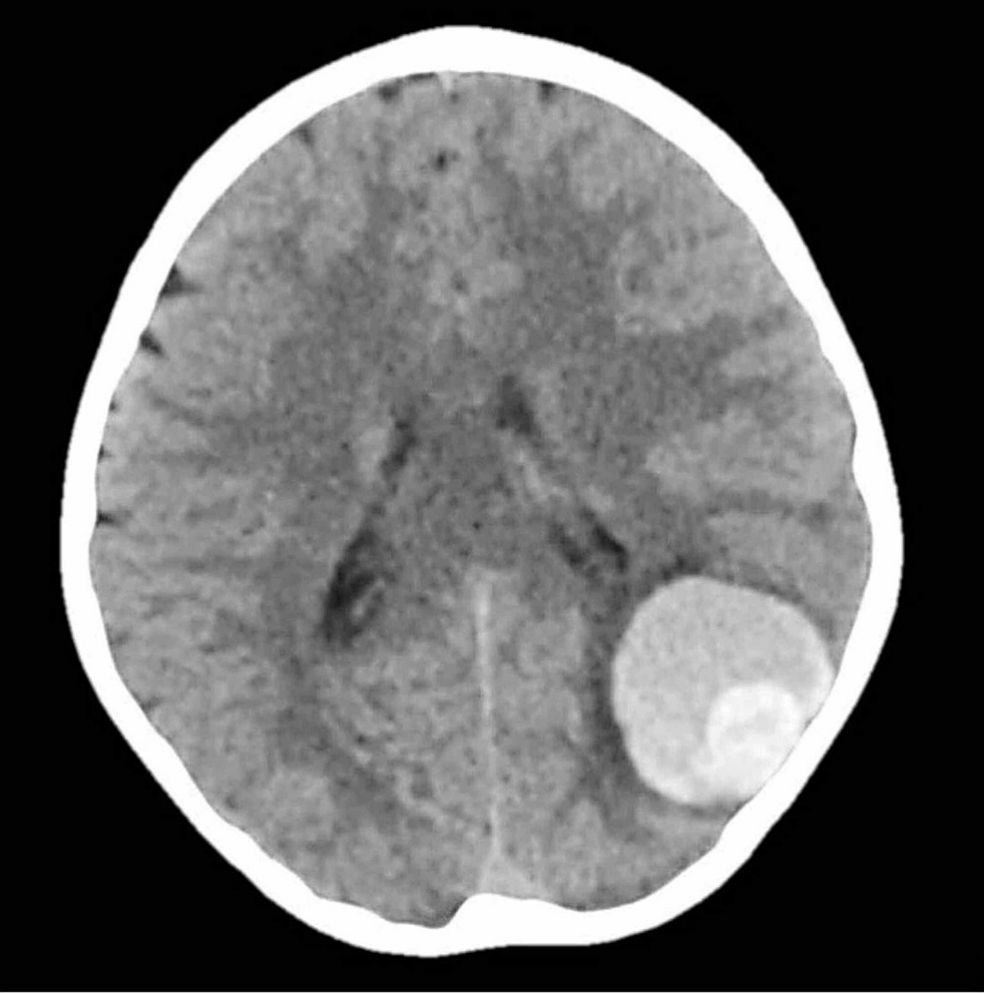
Axial brain CT scan. Axial brain CT scan on admission showing a voluminous hemorrhage in the left temporo-occipital area.

An eight-year-old Bahraini girl, with no known medical illness, was presented to the ED by her parents following a sudden severe headache associated with persistent vomiting. The parents did not report any history of trauma or seizures. She had no significant past medical or surgical history. There was no family history of similar problems or any other neurological defects. On general inspection, she was conscious with a Glasgow Coma Scale (GCS) of 15 out of 15, alert and oriented to time, person, and place. The vitals were as follows: pulse rate 110 beats per minute, blood pressure 110/60 mmHg, O2 saturation 99%, and the temperature was 37.6°C. Neurological examinations such as extraocular movements, pupillary reflex, and cranial nerves assessment were normal. Her higher mental functions were also normal. No papilloedema was noted. All limb motor functions, as well as cerebellar functions and gait, were normal. Examination of other systems was also unremarkable except for a mild abdominal tenderness in the upper part. A brain CT scan was obtained. The pre-contrast CT demonstrated a large round well-defined mixed high-density area in the left parietooccipital region suggesting hemorrhage surrounded by a rim of edema causing adjacent sulci effacement (Figure 1).

**Figure 2:**
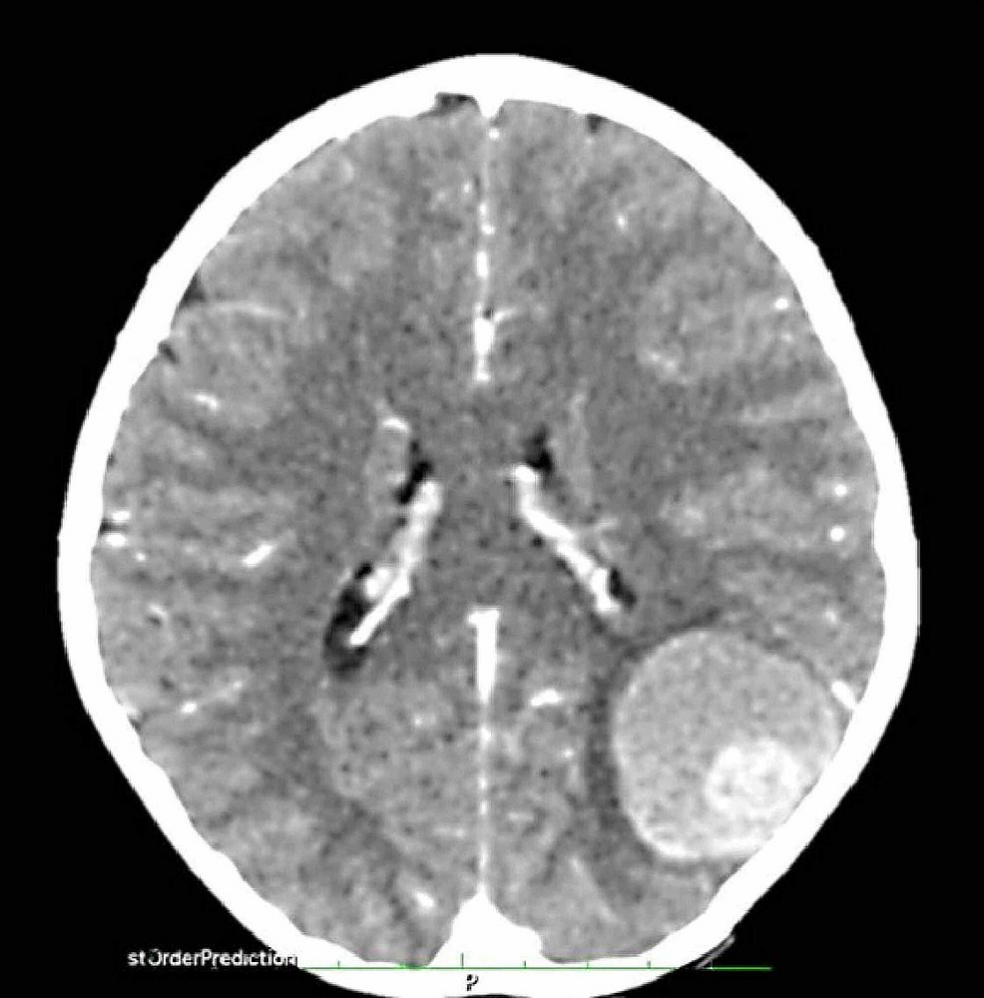
CT angiography image. Normal meningeal vessel enhancement can be seen. No obvious feeding artery was detected.

Post-contrast images showed normal meningeal and cerebral vessel enhancement (Figure 2).

**Figure 3:**
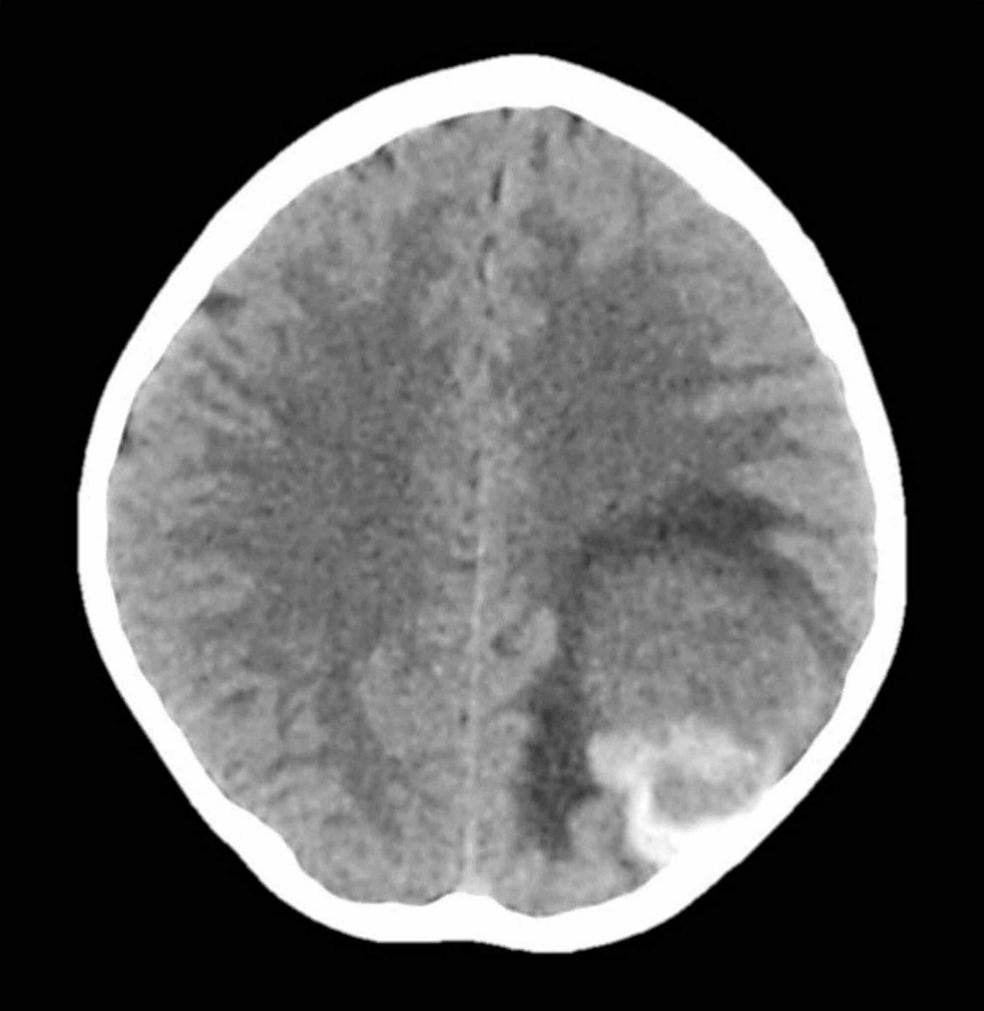
Urgent CT scan of the brain. The urgent CT scan revealed rebleeding in the previous hemorrhage site with signs of resorption of the first intracranial hemorrhage.

No obvious aneurysmal dilatation or feeding artery was noted at the site of hemorrhage. The CT showed normal grey-white matter differentiation, no midline shift, patent basal cisterns, and unremarkable posterior fossa. The impression was a left parietooccipital hemorrhage, possibly due to a cerebral vascular anomaly. The four-vessel angiography demonstrated a mass effect in the left parietal region with no increased vascularity noted in the region of the hematoma. Pre-contrast images of an urgent MRI showed a well-circumscribed rounded area of mixed-signal intensity at the supratentorial compartment located at the left parietooccipital region and close to the dural margin. The size was about 4 x 4 x 4 cm. No significant mass effect was seen. Post-contrast images showed no significant enhancement. The impression was suggestive of acute or subacute bleeding at the left parietooccipital region. Following the investigations, the patient was admitted for close observation and monitoring. She was kept on analgesic, anticonvulsants, and GI prophylaxis. Seven days later, the patient developed a severe headache with vomiting. An urgent CT was performed. It showed the previous large round well-defined lesion has increased in size and the vasogenic edema appeared to be more prominent than the previous images (Figure 3).

**Figure 4:**
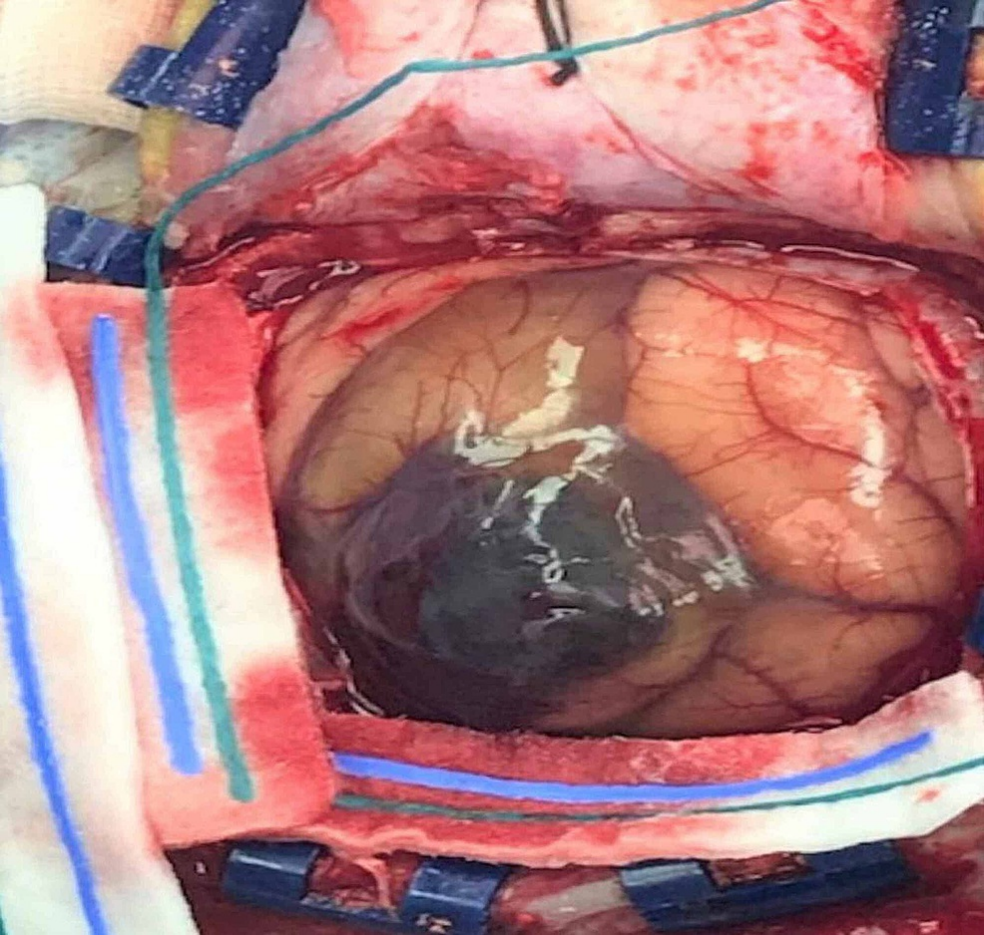
Gross appearance of the lesion. The gross appearance of the lesion was consistent with venous cavernoma (mulberry appearance with gliotic changes around it), filled with clots.

There was a new 3.5 cm midline shift at the level of the septum pellucidum and third ventricle. The impression was similar to the previous radiological studies. The digital subtraction angiography (DSA) was done the next day and no vascular anomaly was seen. Subsequently, the patient was planned for surgical evacuation of the hematoma and exploration for any lesion. She underwent left parietal craniotomy and evacuation of the hematoma and lesion under general anesthesia with the assistance of a NeuroNavigator. Operatively, the gross appearance of the lesion was consistent with venous cavernoma (mulberry appearance with gliotic changes around it) filled with clots (Figure 4).

**Figure 5:**
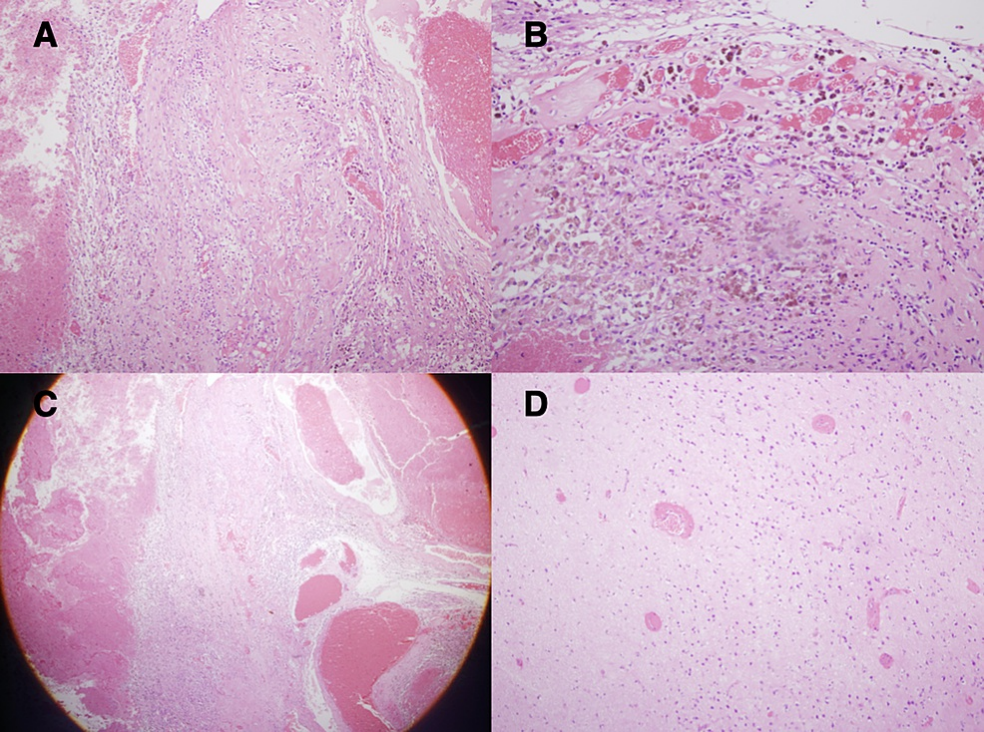
The histopathology slides.

Biopsy was taken to the histopathology laboratory. The report explained that the sections of the entire specimen illustrated blood clots and dilated thin-walled blood vessels, some of which are thrombosed, while others reveal a distinct layer of foamy histiocytes/siderophages within and around. Patchy calcification is observed in some of these blood clots. No specific granulomatous inflammation or any atypical changes detected. Immunohistochemistry findings revealed positive cluster differentiation 68 (CD68) in the foamy histiocytes and negative glial fibrillary acidic protein (Figure 5).

**Figure 6:**
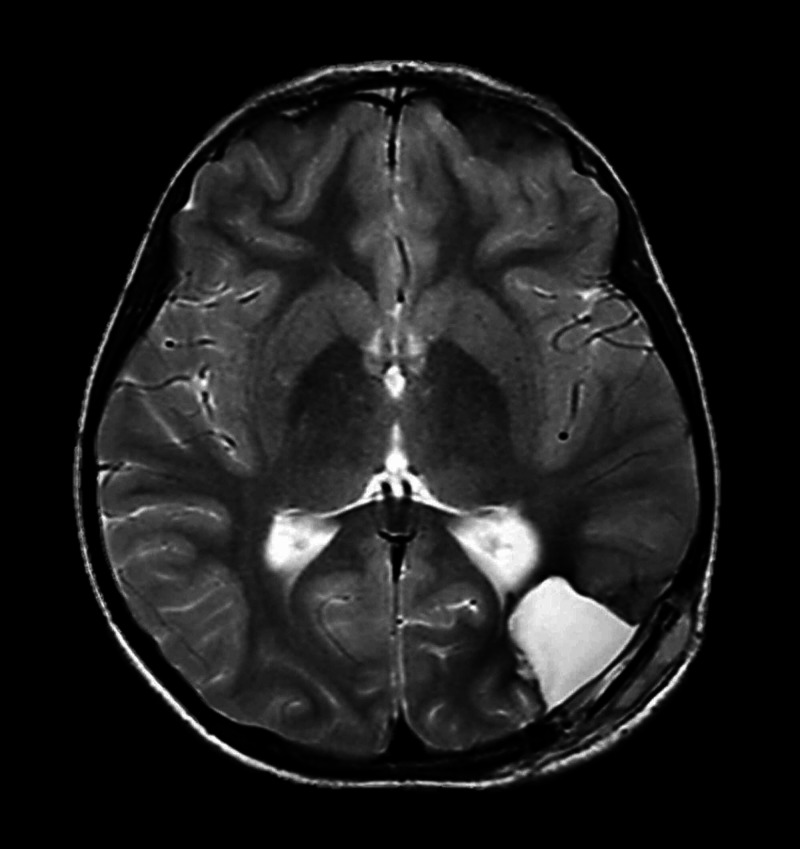
Postoperative axial T2-weighted MRI. Postoperative axial T2-weighted MRI demonstrates evacuation of hematoma and removal of the causative cavernous malformation.

Postoperatively, she was extubated, kept in the pediatric intensive care unit (PICU) for two days, discharged to the general ward, stayed for three days, and discharged home. On the follow-up a week later, she was asymptomatic, conscious, alert, and oriented with no focal neurological deficits (FNDs). She was advised to continue on anticonvulsant and planned to repeat MRI six months later (Figure 6).

## Discussion

The incidence of CCMs is mainly investigated in adults. However, some data has been published regarding their incidence and prevalence in the pediatric age group. The development of CCMs appears to increase with age and reaching to its plateau at the age of 18 to 20. Collecting data regarding sex distribution from 13 independent studies indicate a male/female ratio of 1.3:1. A total of 359 (57%) of the cases were male out of a total number of 629 pediatric patients with CCMs [1-13]. Moreover, it has a prevalence of 0.2% in infancy and a general prevalence of 0.6% among children age group [14].

Cavernous malformations (CMs; also called cavernous angiomas, cavernous hemangiomas, or cavernomas) are capillary malformations in the brain that can happen sporadically or in a familial pattern. These lesions have an evolutionary nature based on serial MRI studies. There are three genetic loci (CCM1, CCM2, and CCM3) responsible for the familial cavernomas that varies among different ethnic groups [13] Clinical signs and symptoms of patients with CCM depend on the site of brain involvement. However, seizure and headache are known to be the most dominant manifestations of CM among patients. In one study consisting of 30 pediatric patients, aged between six months and 17 years old, all patients were asymptomatic before presenting with seizures in 53.3% and sudden onsets or rapidly progressing headaches among 50% of all study population, followed by symptoms including FND, sixth cranial nerve palsy, ataxia, syncopal episodes, unsteadiness of gait, nausea, and vomiting [2, 4, 5, 7]. Other disseminated studies on pediatric patients with CCMs have shown different symptoms such as loss of consciousness or gaze deviation. It is noteworthy that CM may make patients more susceptible to intracerebral hemorrhage (ICH) and its following symptoms [15].

The diagnosis of CM is made by MRI scan. Brain MRI is the most sensitive and specific imaging modality to reach to definite diagnosis of CM. Recommended sequences include standard T1, T2, susceptibility-weighted imaging (SWI), or gradient recall echo images (if SWI not available). A T1 sequence with gadolinium contrast may be useful in determining if there is an associated developmental venous anomaly (DVA) and to rule out the other forms of masses in the brain. With regard to the diagnosis, angiography may not be helpful and that is because blood flow in the cavernoma lesions is relatively poor. MRI is a better diagnostic tool in this setting. MRI can show a “popcorn” appearance on both T1- and T2-weighted images and the hemosiderin ring around the lesion is best seen on T2 images, indicative of a distant hemorrhage. Once the CM is identified, a contrast-enhanced MRI should be obtained to rule out the presence of DVAs [16]. CT scan often demonstrates an irregular, hyperdense mass with some calcification. With regard to the treatment, those CM lesions which present asymptomatically should be observed regardless of their locations. Once the diagnosis of CCM has been determined, future studies in follow-up may not require imaging with contrast [17]. The characteristic radiographic appearance, if associated with CCM's special symptoms or family history can be a diagnostic method. However, in some cases, the imaging may not be typical, potentially mimicking an alternative etiology. If imaging is atypical, additional diagnostic methods such as genetic studies, pathology reports, or repeat MRIs within four to eight weeks may help to achieve a correct diagnosis. In patients with multiple hemorrhagic lesions that the etiology is not clear and biopsy is not possible, genetic testing can be useful. In addition, in patients where the inherited form of CCM is suspected, genotyping and also a three-generation family tree, assessing for neurologic symptoms should be obtained [17]. The differential diagnoses of CCM consist of several types of hemorrhagic, calcified, or mass-like lesions. Hemorrhagic neoplasms, including primary brain tumors and metastases, may be mistaken for CCMs. CCMs should also be distinguished from other forms of vascular malformations, including arteriovenous malformation (AVM) and capillary malformations that may rarely be similar to CCM. The presence of other types of calcified lesions such as granuloma lesions must be considered in CCM differential diagnosis [18].

Studies have indicated that the size of the lesion cannot predict the risk of repeat hemorrhages [4, 9]. In a retrospective study which was aimed to observe postoperative seizure outcomes in 22 children with CCM, the authors concluded that earlier surgery plans in symptomatic CCM cases will lead to a better prognosis in patients [5].

A decision on how to plan treatment for CCM patients depends on a multitude of factors that are basically related to their symptoms and the risk of secondary seizure or hemorrhage. Stereotactic radiosurgery, microsurgical resection, and conservative therapy are the three methods of treatment for CM lesions. The indications for microsurgical resection of CM are basically characterized by: multiple hemorrhages in eloquent areas or a single hemorrhage in a non-eloquent area that is associated with FND and severe cardiac or respiratory symptoms that may be attributed to patients with CCMs. On the other hand, patients with no signs or symptoms who are diagnosed on MRI should be managed conservatively [19]. According to a study, the complication risks associated with surgical resection depend on the location of the CM lesion [20]. So that, patients’ overall neurological condition was good or excellent in 89.7% of all cases, including in 100% of patients with cranial nerve lesions, 97% with lobar lesions, 87.5% with cerebellar lesions, 75% with spinal cord lesions, and in 64% of those with brainstem lesions [20].

## Conclusions

Cerebral cavernoma mostly presents itself with seizures, headache, and focal deficits. Emergency neurosurgical intervention is rare in pediatric CM cases because they are less likely to cause life-threatening hemorrhages. However, physicians must be prepared for any unforeseen events in cases AVM is suspected. Surgery still remains the gold standard for intracranial cavernoma. Conservative therapy is appropriate for cavernomas that are asymptomatic and for those who had one non-devastating episode of hemorrhage. Complications can occur and extensive informed consent should be obtained. Careful radiological studies and close clinical follow-up should be planned for the patient to monitor the residual or recurrence of the disease.
